# Serum anti-AP3D1 antibodies are risk factors for acute ischemic stroke related with atherosclerosis

**DOI:** 10.1038/s41598-021-92786-9

**Published:** 2021-06-29

**Authors:** Shu-Yang Li, Yoichi Yoshida, Eiichi Kobayashi, Masaaki Kubota, Tomoo Matsutani, Seiichiro Mine, Toshio Machida, Yoshiro Maezawa, Minoru Takemoto, Koutaro Yokote, Yoshio Kobayashi, Hirotaka Takizawa, Mizuki Sata, Kazumasa Yamagishi, Hiroyasu Iso, Norie Sawada, Shoichiro Tsugane, Sohei Kobayashi, Kazuyuki Matsushita, Fumio Nomura, Hisahiro Matsubara, Makoto Sumazaki, Masaaki Ito, Satoshi Yajima, Hideaki Shimada, Katsuro Iwase, Hiromi Ashino, Hao Wang, Kenichiro Goto, Go Tomiyoshi, Natsuko Shinmen, Rika Nakamura, Hideyuki Kuroda, Yasuo Iwadate, Takaki Hiwasa

**Affiliations:** 1 Department of Neurological Surgery, Graduate School of Medicine, Chiba University, Inohana 1-8-1, Chuo-ku, Chiba, 260-8670 Japan; 2 Comprehensive Stroke Center, Chiba University Hospital, Chiba, 260-8677 Japan; 3 Department of Neurological Surgery, Chiba Prefectural Sawara Hospital, Chiba, 287-0003 Japan; 4 Department of Neurological Surgery, Chiba Cerebral and Cardiovascular Center, Chiba, 290-0512 Japan; 5 Department of Neurosurgery, Eastern Chiba Medical Center, Chiba, 283-8686 Japan; 6 Department of Endocrinology, Hematology and Gerontology, Graduate School of Medicine, Chiba University, Chiba, 260-8670 Japan; 7 Department of Diabetes, Metabolism and Endocrinology, School of Medicine, International University of Health and Welfare, Chiba, 286-8686 Japan; 8 Department of Cardiovascular Medicine, Graduate School of Medicine, Chiba University, Chiba, 260-8670 Japan; 9 Port Square Kashiwado Clinic, Kashiwado Memorial Foundation, Chiba, 260-0025 Japan; 10 Department of Public Health Medicine, Faculty of Medicine, University of Tsukuba, Tsukuba, 305-8575 Japan; 11 Department of Preventive Medicine and Public Health, Keio University School of Medicine, Tokyo, 160-8582 Japan; 12 Department of Public Health, Social Department of Social and Environmental Medicine, Graduate School of Medicine, Osaka University, Osaka, 565-0871 Japan; 13 Epidemiology and Prevention Group, Center for Public Health Sciences, National Cancer Center, Tokyo, 104-0045 Japan; 14 Department of Laboratory Medicine and Division of Clinical Genetics, Chiba University Hospital, Chiba, 260-8677 Japan; 15 Department of Medical Technology and Sciences, School of Health Sciences at Narita, International University of Health and Welfare, Chiba, 286-8686 Japan; 16 Division of Clinical Genetics, Chiba Foundation for Health Promotion & Disease Prevention, Chiba, 261-0002 Japan; 17 Department of Frontier Surgery, Graduate School of Medicine, Chiba University, Chiba, 260-8670 Japan; 18 Department of Gastroenterological Surgery and Clinical Oncology, Toho University Graduate School of Medicine, Tokyo, 143-8541 Japan; 19 Department of Biochemistry and Genetics, Graduate School of Medicine, Chiba University, Chiba, 260-8670 Japan; 20 Department of Anesthesia, The First Affiliated Hospital, Jinan University, Guangzhou, 510632 Guangdong People’s Republic of China; 21 Medical Project Division, Research Development Center, Fujikura Kasei Co., Saitama, 340-0203 Japan

**Keywords:** Diagnostic markers, Stroke

## Abstract

Atherosclerosis has been considered as the main cause of morbidity, mortality, and disability worldwide. The first screening for antigen markers was conducted using the serological identification of antigens by recombinant cDNA expression cloning, which has identified adaptor-related protein complex 3 subunit delta 1 (AP3D1) as an antigen recognized by serum IgG antibodies of patients with atherosclerosis. Serum antibody levels were examined using the amplified luminescent proximity homogeneous assay-linked immunosorbent assay (AlphaLISA) using a recombinant protein as an antigen. It was determined that the serum antibody levels against AP3D1 were higher in patients with acute ischemic stroke (AIS), transient ischemic attack, diabetes mellitus (DM), cardiovascular disease, chronic kidney disease (CKD), esophageal squamous cell carcinoma (ESCC), and colorectal carcinoma than those in the healthy donors. The area under the curve values of DM, nephrosclerosis type of CKD, and ESCC calculated using receiver operating characteristic curve analysis were higher than those of other diseases. Correlation analysis showed that the anti-AP3D1 antibody levels were highly associated with maximum intima-media thickness, which indicates that this marker reflected the development of atherosclerosis. The results of the Japan Public Health Center-based Prospective Study indicated that this antibody marker is deemed useful as risk factors for AIS.

## Introduction

According to the reports presented to date, atherosclerosis, a chronic inflammatory injury of the arterial wall, may lead to the complications including acute ischemic stroke (AIS) and cardiovascular disease (CVD)^1–3^. Thus, atherosclerosis has been identified as the leading cause of morbidity and mortality worldwide^4,5^. It was reported that diabetes mellitus (DM) and chronic kidney disease (CKD) have causal roles in induction of atherosclerosis^6,7^. Atherosclerosis and cancer share many ethiological and mechanistical processes^8^. Thus, these atherosclerosis-related diseases including AIS, CVD, DM, CKD, and cancer are interrelated more or less with each other. Although many risk factors and biomarkers have been identified for these atherosclerosis-related diseases^9,10^, their specificity and commonality remain obscure.

Serological analysis of recombinant cDNA expression libraries (SEREX)^11,12^ is one of the most effective and comprehensive methods to identify antigenic targets for various types of malignant tumors in humans; in fact, it has been used to determine more than 1000 novel tumor antigens^11,12^. In this study, we were able to find that the serum antibody levels against some of the SEREX antigens were elevated in patients compared with healthy donors (HDs) and reported TROP2, SLC2A1, TRIM21, and myomegalin as antibody biomarkers for esophageal squamous cell carcinoma (ESCC)^13–16^. SEREX was also introduced in screening the biomarkers for atherosclerosis and identified antibody biomarkers such as RPA2^17^, PDCD11^18,19^, MMP1, CBX1, CBX5^20^, DNAJC2^21^, ASXL2^22^, and LRPAP1^23^ for atherosclerotic diseases including AIS and CVD. Notably, the antibody levels against DHPS, ATP2B4, BMP-1, ASXL2, and LRPAP1 were also elevated in patients with ESCC, which suggests the presence of multiple common biomarkers for atherosclerosis and cancer.

In this study, using the SEREX method screening, adaptor-related protein complex 3 subunit delta 1 (AP3D1) was identified as a target antigen recognized by serum IgG antibodies in the sera of patients with atherosclerosis. Next, to evaluate the specificity and commonality among atherosclerosis-related diseases, the levels of serum anti-AP3D1 antibodies in patients with AIS, DM, CVD, CKD and solid cancer were examined.

## Methods

### Patients and HDs' sera

This study was approved by the Local Ethical Review Board of Chiba University, Graduate School of Medicine (Chiba, Japan) and the review boards of the participating hospitals. All experimental procedures were performed in accordance with the Declaration of Helsinki.

Blood samples were collected from patients who had provided their informed consent. Each serum sample was centrifuged at 2000×*g* for 10 min at 4°C, and the supernatant was stored at −80°C until use. Repeated thawing and the freezing of samples were avoided.

Serum samples of patients with AIS, transient ischemic attack (TIA), deep and subcortical white matter hyperintensity (DSWMH), asymptomatic cerebral infarction (asymptCI), and chronic-phase cerebral infarction (cCI) were provided by the Chiba Prefectural Sawara Hospital, Chiba Rosai Hospital, and Chiba Aoba Municipal Hospital, and sera of patients with DM and CVD were obtained from Chiba University Hospital. The stroke subtype of each patient was determined according to the criteria of the Trial of Org 10,172 in Acute Stroke Treatment classification system^24^. In this study, large-artery atherosclerosis or small-artery occlusion (lacune) were included as AIS or cerebral infarction. Serum samples associated with AIS, TIA, and CVD were obtained within 2 weeks after disease onset. Sera of CKD patients were obtained from the Kumamoto cohort^25,26^, and Chiba University Hospital provided the serum samples of patients with ESCC and colorectal carcinoma (CRC). Sera of HDs were obtained from Chiba University Hospital, Port Square Kashiwado Clinic, and Chiba Prefectural Sawara Hospital. Sample of HDs from Port Square Kashiwado Clinic and Chiba Prefectural Sawara Hospital were selected from individuals who exhibited no abnormalities in cranial magnetic resonance imaging.

### Immune screening: serological identification of antigens by recombinant cDNA expression cloning

Initial screening was conducted using the SEREX method as has been described previously^13–17,27–29^. Sera of patients with atherosclerosis were used to search for antigens that could be recognized by the serum IgG antibody. The library used was a Uni-ZAP XR cDNA phage library containing a human microvascular endothelial cell cDNA library (Stratagene; Agilent Technologies, Inc., La Jolla, CA), which was infected into *Escherichia coli* (*E. coli*) XL1-Blue MRF′. Proteins were then transferred onto nitrocellulose membranes [NitroBind, Osmonics Inc., Minnetonka, MN)], which were pretreated with 10 mM isopropyl-β-D-thiogalactoside (IPTG) (Wako Pure Chemicals, Osaka, Japan) for 30 min. The membranes were blocked for 1 h with 1% protease-free bovine serum albumin (Wako Pure Chemicals), 20 mM Tris–HCl (pH 7.5), 0.15 M NaCl, and 0.05% Tween-20, treated overnight to 1:2000 diluted sera from the patients, and then incubated for 1 h with 1:5000 diluted alkaline phosphatase-conjugated goat anti-human IgG (Jackson ImmunoResearch Laboratories, Inc., West Grove, PA). Finally, using a color development solution [0.3 mg/ml nitroblue tetrazolium (Wako Pure Chemicals), 0.15 mg/ml 5-bromo-4-chloro-3-indolyl-phosphate (Wako Pure Chemicals), 100 mM Tris–HCl (pH 9.5), 100 mM NaCl, and 5 mM MgCl_2_], the positive reactions could be identified. The resulting positive antibody were then re-cloned twice in order to obtain monoclonality^13–17,27–29^.

### Sequence analysis of identified clones

Monoclonal phage cDNA clones were converted to pBluescript phagemids by in vivo excision using the ExAssist helper phage (Stratagene; Agilent Technologies, La Jolla, CA). Plasmid DNA was obtained from the *E. coli* SOLR strains transformed by the phagemids. Homology search of the inserted and sequenced cDNAs using a public database provided by the the National Center for Biotechnology Information (https://blast.ncbi.nlm.nih.gov/Blast.cgi) identified the genes.

### AP3D1 protein expression, extraction, and purification

The region of 2490–4347 of the *AP3D1* gene was isolated and was recombined into the *Eco*RI/*Not*I site of pGEX-4T-1 (GE Healthcare Life Sciences, Pittsburgh, PA), followed by confirmation by DNA sequencing. The *E. coli* BL-21 transfected with pGEX-4T-1-AP3D1 was then treated with 0.1 mM IPTG at 37°C for 3 h to induce the expression of cDNA products. Then, the cells were lysed in BugBuster Master Mix (Merck KGaA, Darmstadt, Germany). With the Glutathione-Sepharose (GE Healthcare Life Sciences, Pittsburgh, PA) column chromatography according to the manufacturer's instructions, glutathione S‑transferase (GST)‑fused-AP3D1 protein was purified, as has been described previously^17,19,20^.

### AlphaLISA (amplified luminescence proximity homogeneous assay-linked immunosorbent assay)

To evaluate the serum antibody levels, AlphaLISA was used. Initially, AlphaLISA was performed in 384-well microtiter plates (white opaque OptiPlate, PerkinElmer, Waltham, MA, USA) containing either 2.5 µl of 1:100 diluted serum or 2.5 µl of 10 µg/ml of GST and GST-AP3D1 protein (25 mM HEPES, pH 7.4, 0.1% casein, 0.5% Triton X-100, 1 mg/ml dextran-500 and 0.05% Proclin-300). The reaction mixture was then incubated at room temperature for 6–8 h. Secondly, after adding anti-human IgG-conjugated acceptor beads (2.5 µl at 40 µg/ml) and glutathione-conjugated donor beads (2.5 µl at 40 µg/ml), the mixture was further incubated at room temperature in the dark for 7–14 days. Chemical emissions were read on an EnSpire Alpha microplate reader (PerkinElmer) as previously described^18–23,30^. Specific reactions were then calculated by subtracting the alpha photon counts of the GST and buffer control from the counts of the GST-AP3D1 protein.

### JPHC cohort analysis

The longitudinal association between plasma AP3D1 levels (using the above AlphaLISA detection antibody levels) and incident AIS were examined in the Japan Public Health Center-based prospective Study (JPHC). The study nested within JPHC cohort^31,32^, involving approximately 30,000 Japanese individuals aged 40–69 years at the baseline period of 1990–1994 whose plasma were stored. The antibody levels of AP3D1 protein were measured in 202 cases of AIS in the cohort developed between the baseline and 2008, and in 202 controls whose sex, age (within 2 years), date of blood sampling (within 3 months), time since last meal (within 4 h) and study location (Public Health Center area) were matched with the cases. We used a conditional logistic regression model to estimate the odds ratios (ORs) and 95% confidence intervals (CIs) for AIS with respect to the antibody levels of AP3D1 protein.

### Statistical analysis

All statistical analyses were conducted using GraphPad Prism 5 (GraphPad Software, La Jolla, CA) and EZR software^33^. The Kruskal–Wallis test (Mann–Whitney *U* test with Bonferroni's correction applied) was used to evaluate differences among > 3 groups, and the Mann–Whitney *U* test was employed to determine significant differences between the two groups. Correlations were calculated using Spearman's correlation analysis and logistic regression analysis. The predictive values of the putative disease markers were assessed using a receiver operating characteristic (ROC) curve analysis, and the cutoff values were set to maximize the sums of sensitivity and specificity. All tests were two-tailed, and *P* values lower than 0.05 were considered to be statistically significant. The power calculation was performed using G-Power 3.1 software (Heinrich-Heine-Universität Düsseldorf).

### Ethics declarations

The present study was approved by the Local Ethical Review Board of Chiba University, Graduate School of Medicine (Chiba, Japan) as well as the review boards of co-operating hospitals or institutes. Serum or plasma was collected from participants who had provided informed consent by following the protocols approved by their institutional ethical committees.

### Consent for publication

Not applicable.

## Results

### Initial screening of AP3D1 antigens using SEREX

As an initial SEREX screening, sera of patients with atherosclerosis were used to search for antigens that could be recognized by serum IgG antibodies, one of which was AP3D1 (accession no. NM_003938.8). The region of 2490–4347 of the *AP3D1* gene was then isolated and recombined into the *Eco*RI/*Not*I site of pGEX-4T-1, followed by confirmation by DNA sequencing. The cDNA was then expressed in *E. coli*, purified by affinity chromatography, and employed as an antigen in order to examine the serum antibody levels.

### The levels of anti-AP3D1 antibodies were elevated in patients with AIS and TIA

**Figure 1:**
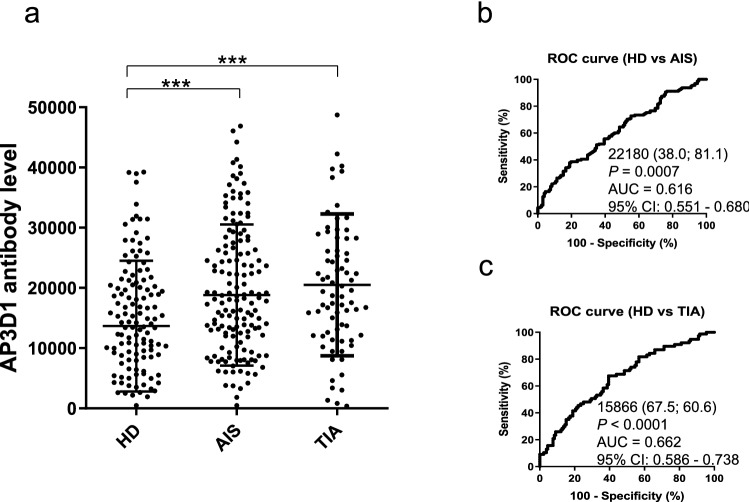
Comparing the serum AP3D1-antibody levels between healthy donors (HDs) and patients with AIS and TIA. This figure shows the levels of serum antibodies against AP3D1 (s-AP3D1-Abs) examined using amplified luminescence proximity homogeneous assay-linked immunosorbent assay (AlphaLISA) (**a**). The bars represent the average and average ± standard deviation (SD). *P*-values were calculated using the Kruskal–Wallis test (Mann–Whitney *U* test with Bonferroni's correction applied). ***P < 0.001. The total average values, SDs, cutoff values, positive numbers, positive rates (%), and *P*-values are summarized in Table 1. A receiver operating characteristic (ROC) curve analysis was performed to assess the abilities of s-AP3D1-Abs in detecting either (**b**) acute ischemic stroke (AIS) or (**c**) transient ischemic attack (TIA). The numbers in the figures indicate the cutoff values for marker levels, and the numbers in parentheses indicate sensitivity (left) and specificity (right). *P* value, area under the curve (AUC), and 95% confidence intervals (95% CI) are also shown. AP3D1, adaptor-related protein complex 3 subunit delta 1. The results of the power calculation using G-Power 3.1 software were 0.9762618 for AIS and 0.9953412 for TIA (Supplementary Table S3).

The serum anti-AP3D1-antibody (s-AP3D1-Ab) levels in patients with AIS and TIA were examined using AlphaLISA. AIS and TIA sera were provided by Chiba Prefectural Sawara Hospital, Chiba Rosai Hospital, and Chiba Aoba Municipal Hospital, whereas samples of HDs were obtained from Chiba University, Port Square Kashiwado Clinic, and Chiba Prefectural Sawara Hospital. The average ages [± standard deviations (SDs)] of the HDs and patients with AIS and TIA were 51.85 ± 8.74, 57.99 ± 7.97, and 69.45 ± 11.64 years, respectively (Table 1 upper panel). The levels of s-AP3D1-Abs were determined to be significantly higher in patients with AIS and TIA than those in HDs (Fig. 1a). When the ages of the subjects were matched to 65 years, the s-AP3D1-Ab levels were still significantly higher in patients with AIS than those in HDs (Supplementary Fig. S1a). At a cutoff value equivalent to the average plus two SDs of the HD values, the s-AP3D1-Ab-positive rates in HDs and patients with AIS and TIA were 2.4%, 10.1%, and 10.4%, respectively (Table 1 lower panel). ROC curve analysis revealed that the area under the curve (AUC) values for s-AP3D1-Abs vs. AIS and vs. TIA were 0.616 and 0.662, respectively (Fig. 1b,c). No significant difference was found in the positive rates and the AUC values between AIS and TIA.

**Table 1:** Comparing the serum antibody levels against AP3D1 between healthy donors (HDs) and patients with AIS and TIA.

Subject information on HDs and patients with AIS and TIA			
Sample information	HD	AIS	TIA
Total number	123	158	77
Male/Female	85/38	119/39	49/28
Age, years (average ± SD)	51.85 ± 8.74	57.99 ± 7.97	69.45 ± 11.64

Summary of serum AP3D1 antibody (s-AP3D1-Ab) levels examined by AlphaLISA in HDs and patients with AIS and TIA			
Patient group	Type of value	s-AP3D1-Ab	
HD	Average	13,471	
	SD	10,800	
	Cutoff values	35,072	
	Positive No	3	
	Positive (%)	2.40%	
AIS	Average	18,810	
	SD	11,712	
	Positive No	16	
	Positive (%)	**10.10%**	
	*P* value (vs. HD)	** < 0.001**	
TIA	Average	20,506	
	SD	11,786	
	Positive No	8	
	Positive (%)	**10.40%**	
	*P* value (vs. HD)	** < 0.001**	

The upper panel indicates the number of total samples, samples from male and female participants, and ages [average ± standard deviation (SD)]. The lower panel summarizes the serum AP3D1 antibodies (s-AP3D1-Abs) examined using amplified luminescence proximity homogeneous assay-linked immunosorbent assay (AlphaLISA) using purified AP3D1 protein as an antigen. Cutoff values were determined as the average HDs values plus two SDs, and positive samples for which the antibody levels exceeded the cutoff value were scored. *P*-values were calculated using the Kruskal–Wallis test (Mann–Whitney *U* test with Bonferroni's correction applied). *P*-values of < 0.05 and positive rates of > 10% are marked in bold font. These data are plotted and shown in Fig. 1a,b.

*AP3D1* adaptor-related protein complex 3 subunit delta 1, *s-AP3D1-Abs* the serum anti-AP3D1 antibodies, *AIS* acute ischemic stroke, *TIA* transient ischemic attack.

### Elevation of s-AP3D1-Abs levels in patients with DM

**Figure 2:**
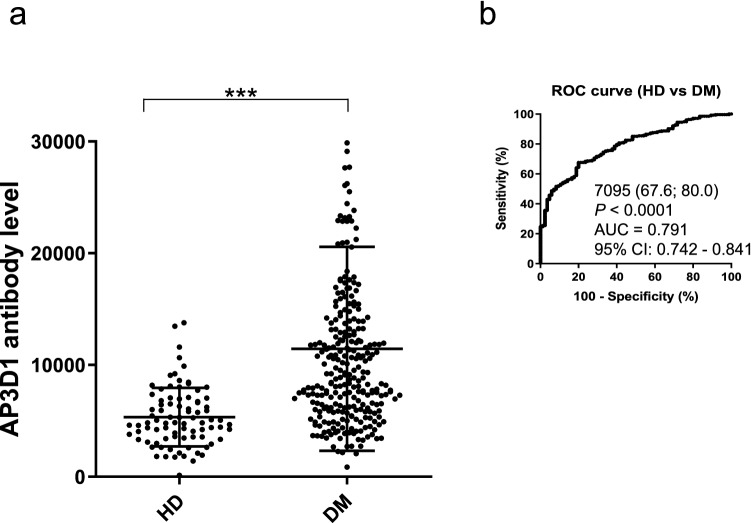
Comparing the levels of serum antibodies against AP3D1 between HDs and patients with DM. The s-AP3D1-Abs levels in HDs and patients with diabetes mellitus (DM) examined using AlphaLISA are shown (**a**). The bars represent the average and average ± SD. *P*-values were calculated using the Mann–Whitney *U* test. ***P < 0.001. The data are summarized in Table 2. ROC curves to assess the ability of s-AP3D1-Abs to predict DM are shown (**b**). The numbers in the figures are the same as those shown in Fig. 1. The results of the power calculation were shown in Supplementary Table S3.

The levels of s-AP3D1-Abs were also examined for DM. Sera of HD were obtained from Chiba University, whereas the sera of patients with DM were provided by the Chiba University Hospital. The average ages (± SDs) of the HDs and patients with DM were 45.20 ± 10.95 and 63.12 ± 12.04 years, respectively. The AlphaLISA results revealed that s-AP3D1-Ab levels were significantly higher in patients with DM than in the HDs (Fig. 2a). When the levels of s-AP3D1-Abs were compared between age-matched (60 years) HDs and patients with DM, the levels were also significantly higher in patients with DM than in HDs (Supplementary Fig. S1b). When the positive samples for which the AlphaLISA counts exceeded the cutoff value were scored, the positive rates of s-AP3D1-Abs in the HDs and the patients with DM were 3.7% and 41.8%, respectively (Table 2). The AUC value of s-AP3D1-Abs vs. DM was as high as 0.791 (Fig. 2b). Therefore, it can be concluded that the s-AP3D1-Ab levels were closely associated with DM.

**Table 2:** Comparing the anti-AP3D1 antibody levels between HDs and patients with diabetes mellitus (DM).

Subject information on HDs and patients with DM			
Sample information		HD	DM
Total sample number		81	275
Male/female		46/35	158/117
Age, years (average ± SD)		45.20 ± 10.95	63.12 ± 12.04

Summary of serum AP3D1 antibody levels (s-AP3D1-Ab) examined by AlphaLISA in HDs and patients with DM			
Patient group	Type of value	s-AP3D1-Ab	
HD	Average	5439	
	SD	2640	
	Cutoff values	10,720	
	Positive No	3	
	Positive (%)	3.70%	
DM	Average	11,450	
	SD	9125	
	Positive No	115	
	Positive (%)	**41.80%**	
	*P* value (vs. HD)	** < 0.001**	

The upper panel indicates the number of total samples, samples from male and female participants, and ages (average ± SD). The lower panel summarizes the s-AP3D1-Ab levels examined using AlphaLISA. Numbers are as shown in Table 1; *P*-values of < 0.05 and positive rates of > 10% are marked in bold font. The plots for these data are shown in Fig. 2b.

### The s-AP3D1-Abs levels were associated with CVD

**Figure 3:**
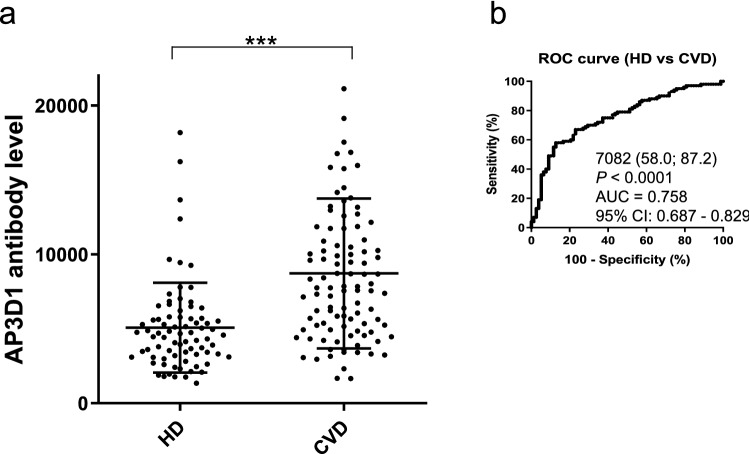
Comparing the serum AP3D1-Ab levels between HDs and cardiovascular disease (CVD) patients. This figure shows the s-AP3D1-Ab levels in HDs and CVD patients examined using AlphaLISA (**a**). The bars represent the average and average ± SD. *P*-values were calculated using the Mann–Whitney *U* test. ***P < 0.001. The data are summarized in Table 3. ROC curves to assess the ability of s-AP3D1-Abs to predict CVD are shown (**b**). The numbers in the figures are the same as those shown in Fig. 1. The results of the power calculation were shown in Supplementary Table S3.

For the next step, the antibody levels in samples from CVD patients were examined. The samples of CVD patients were obtained from Chiba University Hospital, and those in HDs were from Chiba University, Port Square Kashiwado Clinic, and Chiba Prefectural Sawara Hospital. The average ages (± SDs) of the HDs and CVD patients were 45.27 ± 11.20 and 66.07 ± 11.32 years, respectively. Compared with HDs, s-AP3D1-Abs levels were significantly higher in patients with CVD (Fig. 3a), and the s-AP3D1-Ab positivity rates in HDs and patients with CVD were 5.1% and 24.0%, respectively (Table 3). ROC curve analysis revealed that AUC of s-AP3D1-Abs for CVD was 0.758 (Fig. 3b).

**Table 3:** Comparing the anti-AP3D1 antibody levels between HDs and cardiovascular disease (CVD) patients.

Subject information on HDs and patients with CVD			
Sample information		HD	CVD
Total sample number		78	100
Male/female		46/32	84/16
Age, years (average ± SD)		45.27 ± 11.20	66.07 ± 11.32

Summary of serum AP3D1 antibody levels (s-AP3D1-Ab) examined by AlphaLISA in HDs and patients with CVD			
Patient group	Type of value	s-AP3D1-Ab	
HD	Average	6307	
	SD	3123	
	Cutoff values	12,553	
	Positive No	4	
	Positive (%)	5.10%	
CVD	Average	10,015	
	SD	5129	
	Positive No	24	
	Positive (%)	**24.00%**	
	*P* value (vs. HD)	** < 0.001**	

The upper panel indicates the number of total samples, samples from male and female participants, and ages (average ± SD). The lower panel summarizes the s-AP3D1-Ab levels examined using AlphaLISA. Numbers are as shown in Table 1; *P*-values of < 0.05 and positive rates of > 10% are marked in bold font. The plots for these data are shown in Fig. 2.

### The s-AP3D1-Ab levels were closely related to CKD

**Figure 4:**
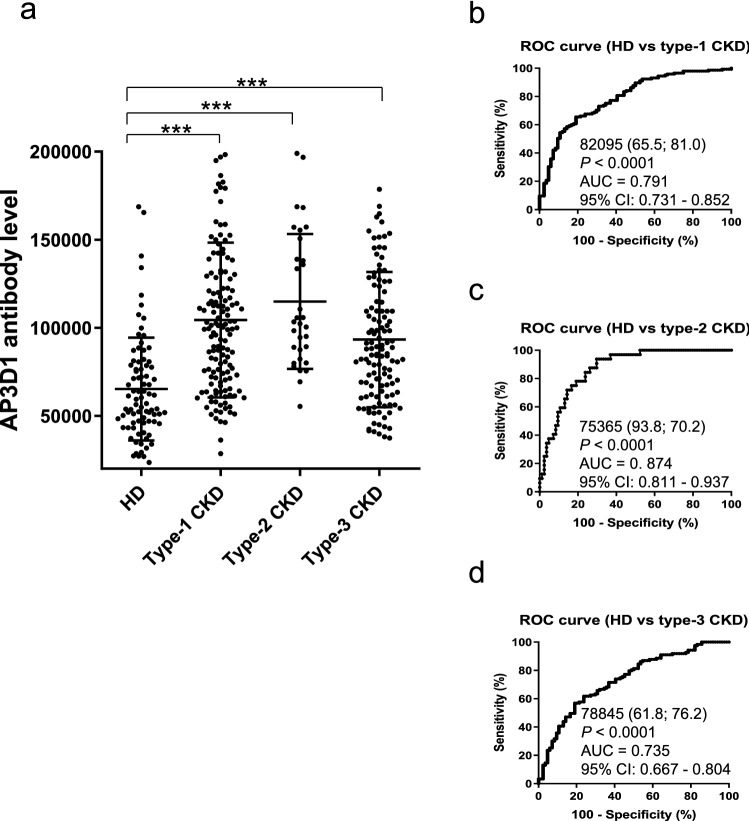
Comparing the serum AP3D1-antibody levels between HDs and patients with chronic kidney disease (CKD). (**a**) The s-AP3D1-Ab levels were compared between HDs and patients with diabetic CKD (type 1 CKD), nephrosclerosis (type 2 CKD), and glomerulonephritis (type 3 CKD). Results are presented as described in the legend of Fig. 1. *P*-values vs. HD controls were calculated using the Kruskal–Wallis test. ***P < 0.001. The data are summarized in Table 4. The total average values, SDs, cutoff values, positive numbers, positive rates (%), and *P*-values are summarized in Table 4. The ability of s-AP3D1-Abs to (**b**) predict type 1, (**c**) type 2, and (**d**) type 3 CKD was also evaluated using the ROC curve analysis. The results of the power calculation were shown in Supplementary Table S3.

The antibody levels in the sera of CKD patients were examined, which were assumed to be closely related to atherosclerosis. The sera of the CKD patients were obtained from the Kumamoto cohort^22,23^, including 145 from patients with diabetic kidney disease (type 1 CKD), 32 from patients with nephrosclerosis (type 2 CKD), and 123 from patients with glomerulonephritis (type 3 CKD). The sera of HDs (82 specimens) were obtained from Chiba University, Chiba Prefectural Sawara Hospital, and the National Hospital Organization of Shimoshizu Hospital. Patients from all three groups of CKD were found to have significantly higher levels of s-AP3D1-Abs compared to that in HDs (Fig. 4a). The s-AP3D1-Ab-positive rates in HDs and patients with type 1, type 2, and type 3 CKD were 4.9%, 27.6%, 37.5%, and 22.8%, respectively (Table 4). ROC curve analysis revealed AUC of s-AP3D1-Abs of type 1, type 2, and type 3 CKD to be 0.791, 0.874, and 0.735, respectively (Fig. 4b–d). Type 2 CKD showed the highest AUC value among all diseases examined.

**Table 4:** Comparing the s-AP3D1-Ab levels between HDs and patients with chronic kidney disease (CKD).

Numbers for the total samples, samples from male and female participants and ages (average ± SD)				
Sample information	HD	Type-1 CKD	Type-2 CKD	Type-3 CKD
Total sample number	82	145	32	123
Male/Female	44/38	106/39	21/11	70/53
Age, years (average ± SD)	44.10 ± 11.19	66.04 ± 10.38	76.03 ± 9.78	61.98 ± 11.69

Serum AP3D1 antibody levels (s-AP3D1-Ab) examined by AlphaLISA				
Patient group	Type of value	s-AP3D1-Ab		
HD	Average	64,142		
	SD	28,679		
	Cutoff values	121,499		
	Positive No	4		
	Positive (%)	4.90%		
Type-1 CKD	Average	104,416		
	SD	43,875		
	Positive No	40		
	Positive (%)	**27.60%**		
	*P* value (vs. HD)	** < 0.001**		
Type-2 CKD	Average	114,921		
	SD	38,248		
	Positive No	12		
	Positive (%)	**37.50%**		
	*P* value (vs. HD)	** < 0.001**		
Type-3 CKD	Average	93,285		
	SD	38,419		
	Positive No	28		
	Positive (%)	**22.80%**		
	*P* value (vs. HD)	** < 0.001**		

The numbers shown are as described in Table 1. CKD was divided into three groups as follows: type 1, diabetic kidney disease; type 2, nephrosclerosis; and type 3, glomerulonephritis. *P* values of < 0.05 and positive rates of > 10% are marked in bold font. The plots for these data are shown in Fig. 4a.

### Association of s-AP3D1-Ab levels with ESCC or CRC

**Figure 5:**
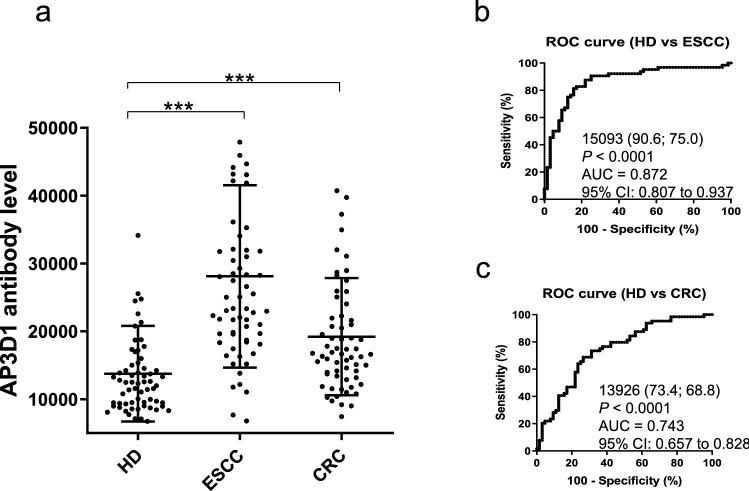
Comparing the serum AP3D1-antibody levels between HDs and patients with digestive organ cancer. This figure shows the (**a**) s-AP3D1-Ab levels in HDs and patients with esophageal squamous cell carcinoma (ESCC) or colorectal carcinoma (CRC) examined using AlphaLISA. The bars represent the average and average ± SD. *P*-values were calculated using the Kruskal–Wallis test. ***P < 0.001. The data are summarized in Table 5. ROC curves to assess the ability of s-AP3D1-Abs to predict (**b**) ESCC and (**c**) CRC are shown. The numbers in the figures are the same as those shown in Fig. 1. The results of the power calculation were shown in Supplementary Table S3.

The s-AP3D1-Abs levels were also measured in serum samples from the HDs and patients with ESCC or CRC. AlphaLISA results revealed that s-AP3D1Ab levels were significantly higher in patients with ESCC and CRC than in HDs (Fig. 5a). The positivity rates of s-AP3D1-Abs in HDs and patients with ESCC and CRC were 3.1%, 42.2%, and 15.6%, respectively (Table 5). The AUC values were 0.872 and 0.743 for ESCC and CRC, respectively (Fig. 5b,c).

**Table 5:** Comparing the serum anti-AP3D1 antibody levels of HDs versus those of patients with esophageal squamous cell carcinoma (ESCC) and colorectal carcinoma (CRC).

Patient group	Type of value	s-AP3D1-Ab
HD	Average	15,169
	SD	7065
	Cutoff values	29,299
	Total No	64
	Positive No	2
	Positive (%)	3.10%
ESCC	Average	29,537
	SD	13,468
	Total No	64
	Positive No	27
	Positive (%)	**42.20%**
	*P* value (vs. HD)	** < 0.001**
CRC	Average	20,639
	SD	8,659
	Total No	64
	Positive No	10
	Positive (%)	**15.60%**
	*P* value (vs. HD)	** < 0.001**

The s-AP3D1-Ab levels examined using AlphaLISA in HDs and patients with ESCC and CRC are shown. Purified AP3D1-GST proteins were used as antigens. The numbers shown are as described in Table 1. *P*-values of < 0.05 and positive rates of > 10% are marked in bold font. The plots for these data are shown in Fig. 5a.

### Correlation analysis

Correlation analysis of s-AP3D1-Ab levels and subject data was performed using 633 specimens from Chiba Prefectural Sawara Hospital, including 139 samples from HDs, 121 from patients with DSWMH, 17 from patients with asymptCI, 43 from patients with TIA, 226 from patients with AIS, 57 from patients with cCI, and 30 from other diseases. Baseline characteristics of the study subjects in Sawara Hospital cohort are summarized in Supplementary Table S1. Using the Mann–Whitney *U* test, the s-AP3D1-Ab levels were compared between participants with body mass index (BMI) < 25 and BMI ≥ 25; participants with or without diseases of DM, hypertension (HT), CVD, and dyslipidemia; and between those patients who were smokers or nonsmokers and those who consumed alcohol or not. The analysis showed that the s-AP3D1-Ab levels were significantly higher in the subjects with HT than in those without HT and those with DM than without DM (Table 6). Conversely, no significant differences in s-AP3D1-Ab levels were observed in the other categories.

**Table 6:** Association between s-AP3D1-Ab levels with data from participants in the Sawara Hospital cohort.

Category	Category division	Category division
Sex	Male	Female
Sample No	396	269
**s-AP3D1-Ab levels**		
Average	11,446	12,628
SD	9468	8592
*P* value (vs. male)		**0.022**

Obesity	BMI < 25	BMI ≥ 25
Sample No	498	167
**s-AP3D1-Ab levels**		
Average	12,065	11,502
SD	9646	7418
*P* value (vs. BMI < 25)		0.783

Other disease	DM^-^	DM^+^
Sample No	525	140
**s-AP3D1-Ab levels**		
Average	11,777	12,475
SD	9707	6564
*P* value (vs. DM^-^)		**0.011**

Other disease	HT^-^	HT^+^
Sample No	239	426
**s-AP3D1-Ab levels**		
Average	10,308	12,830
SD	9547	8777
*P* value (vs. HT^-^)		** < 0.0001**

Other disease	CVD^-^	CVD^+^
Sample No	623	42
**s-AP3D1-Ab levels**		
Average	11,770	14,212
SD	9073	9854
*P* value (vs. CVD^-^)		0.098

Other disease	Lipidemia^-^	Lipidemia^+^
Sample No	480	185
**s-AP3D1-Ab levels**		
Average	12,352	10,814
SD	9907	6629
*P* value (vs. lipidemia^-^)		0.17

Lifestyle	Non-smoker	Smoker
Sample No	346	319
**s-AP3D1-Ab levels**		
Average	11,131	12,785
SD	7370	10,672
*P* value (vs. non-smoker)		0.06

Lifestyle	Alcohol^−^	Alcohol^+^
Sample No	238	427
**s-AP3D1-Ab levels**		
Average	12,529	11,587
SD	8592	9417
*P* value (vs. alcohol^−^)		0.064

The participants were divided as follows: sex (male and female), obesity [body mass index (BMI)], presence (+) or absence (−) of DM complications, hypertension (HT), CVD or dyslipidemia, and lifestyle factors (smoking and alcohol intake habits). The s-AP3D1-Ab levels divided into two groups were compared using the Mann–Whitney *U* test. Sample numbers, averages, and SDs of the counts and the *P*-values are shown. Significant associations (*P* < 0.05) are marked in bold font.

Performing a logistic regression analysis of the predictors for AIS using the results of the Sawara Hospital was also considered, which included 139 samples from HDs and 226 from patients with AIS. An elevated s-AP3D1-Ab level was associated with an increased risk of AIS as shown by the univariate logistic regression analysis (*P* < 0.0001). A multivariate logistic regression analysis has identified age, HT, and DM, but not s-AP3D1-Ab, as independent predictors of AIS (Supplementary Table S2).

Next, correlation analysis was performed using a Spearman application in order to determine the correlation between s-AP3D1-Ab levels and subject parameters, including general information such as age, body height, weight, BMI, and the degree of artery stenosis [the maximum intima-media thickness (max IMT)]. The serum s-AP3D1-Ab levels were determined to be closely correlated with age　(*P* < 0.001), max IMT (*P* < 0.001), blood pressure (BP) (*P* < 0.001), and smoking period (*P* < 0.001**)** (Table 7). Conversely, inverse correlation was observed between s-AP3D1-Ab levels and height, weight, Ca, and low-density lipoprotein cholesterol. Blood glucose and glycated hemoglobin, which are identified as DM markers, were not significantly correlated with the s-AP3D1-Abs levels. These results suggest that s-AP3D1-Ab reflected atherosclerosis and its causal HT and smoking.

**Table 7:** Correlation analysis of the s-AP3D1-Ab levels with data on participants in the Sawara Hospital cohort.

	*r* value	*P* value
Age	0.268	** < 0.001**
Height (cm)	− 0.206	** < 0.001**
Weight (kg)	− 0.158	** < 0.001**
BMI	− 0.046	0.240
max IMT	0.226	** < 0.001**
A/G	− 0.047	0.237
AST (GOT)	0.039	0.317
ALT (GPT)	0.006	0.876
ALP	0.070	0.088
LDH	0.061	0.128
tBil	0.008	0.846
CHO	− 0.063	0.156
TP	0.004	0.926
ALB	− 0.034	0.385
BUN	− 0.045	0.251
Creatinin	− 0.046	0.244
eGFR	0.045	0.294
UA	− 0.038	0.405
T-CHO	− 0.081	0.053
HDL-c	− 0.032	0.511
TG	− 0.044	0.341
K	− 0.063	0.110
Cl	− 0.016	0.682
Ca	− 0.102	**0.046**
IP	− 0.014	0.815
Fe	− 0.023	0.683
CRP	0.072	0.116
LDL-C	− 0.107	**0.047**
WBC	0.065	0.098
RBC	− 0.022	0.577
HGB	− 0.018	0.648
HCT	− 0.015	0.703
MCV	0.037	0.340
MCH	0.002	0.965
MCHC	− 0.040	0.305
RDW	0.054	0.166
PLT	− 0.012	0.755
MPV	− 0.028	0.476
PCT	− 0.006	0.888
BS	0.078	0.058
HbA1c	0.005	0.918
BP	0.145	** < 0.001**
Smoking period	0.141	** < 0.001**
Alcohol frequency	− 0.065	0.096

Correlation coefficients (*r* values) and *P* values obtained using Spearman's correlation analysis are shown. Significant correlations (*P* < 0.05) are marked in bold font. max IMT, maximum intima-media thickness; A/G, albumin/globulin ratio; AST, aspartate aminotransferase; ALT, alanine aminotransferase; ALP, alkaline phosphatase; LDH, lactate dehydrogenase; tBil, total bilirubin; CHO, cholinesterase; TP, total protein; ALB, albumin; BUN, blood urea nitrogen; creatinine, eGFR, estimated glomerular filtrating ratio; UA, uric acid; T-CHO, total cholesterol; HDL-C, high-density lipoprotein cholesterol; TG, triglyceride; K, potassium; Cl, chlorine; Ca, calcium; IP, inorganic phosphate; Fe, iron; CRP, C-reactive protein; LDL-C, low-density lipoprotein cholesterol; WBC, white blood cell; RBC, red blood cell; HGB, hemoglobin; HCT, hematocrit; MCV, mean corpuscular volume; MCH, mean corpuscular hemoglobin; MCHC, mean corpuscular hemoglobin concentration; RDW, red cell distribution width; PLT, platelet; MPV, mean platelet volume; PCT, procalcitonin; BS, blood sugar; HbA1c, glycated hemoglobin; BP, blood pressure.

### JPHC cohort analysis

To determine whether s-AP3D1-Ab marker can be applied to predict the onset of AIS, JPHC cohort samples were examined. The antibody level of AP3D1 protein was positively and strongly associated with the risk of AIS: the ORs (95% CI) were 1.40 (0.75–2.63), 1.97 (1.07–3.65), and 2.28 (1.26–4.13) for the samples with the second, third, and highest quartiles of antibody level, respectively, compared with the lowest quartile (Table 8). These results indicate that the antibody markers against AP3D1 are useful in predicting the onset of AIS.

**Table 8:** Results of JPHC cohort subjects.

	Case/control	Matched OR (95% CI)
**AP3D1-Ab vs AIS**		
2nd	40/50	1.40 (0.75–2.63)
3rd	59/51	1.97 (1.07–3.65)
4th	71/50	2.28 (1.26–4.13)

Age-, sex-, and area-matched, conditional odds ratios, and 95% confidence intervals of AIS according to AP3D1 antibody markers.

*OR* odds ratios.

## Discussion

Through the initial SEREX screening, AP3D1 was identified as an antigen recognized by serum IgG in patients with atherosclerosis. The s-AP3D1-Ab levels were higher in patients with AIS, TIA, DM, CVD, CKD, ESCC, and CRC than in the HDs (Figs. 1–5 and Tables 1–5). Meanwhile, to reduce the effect of age, we compared the serum antibody levels of HDs and age-matched patients. The results showed that the s-AP3D1-Ab levels in patients with AIS and DM were significantly higher than those in HDs (Supplementary Fig. S1). Among these diseases, the highest positive rates were observed for ESCC, DM, and type 2 CKD (Tables 1–5). The AUC values for nephrosclerosis type 2 CKD and ESCC, diabetic type 1 CKD, and DM were 0.874 and 0.872, 0.791, and 0.791, respectively, which were higher than other diseases. The comparison using the Mann–Whitney *U* test revealed that the s-AP3D1-Ab levels were significantly higher in the subjects with DM than in those without DM (Table 6). In contrast, no significant correlation was found between the s-AP3D1-Ab levels and DM markers, including blood glucose and glycated hemoglobin (Table 7). Consequently, the s-AP3D1-Ab levels do not directly reflect DM, but are associated with DM-induced atherosclerotic disorders, which are also related to CKD and cancer. Consistently, Spearman correlation analysis revealed a significant association between s-AP3D1-Ab levels and max IMT (*P* < 0.001), which reflects arterial stenosis, namely, atherosclerosis (Table 7). The antibody levels significantly correlated with HT (*P* < 0.0001) (Table 6), which are well-known risk factors for atherosclerosis^34^. A univariate logistic regression analysis revealed that an elevated AP3D1‑Ab level was associated with an increased risk of AIS (*P* < 0.0001). A multivariate logistic regression analysis has also identified age, HT, and DM, but not AP3D1‑Ab as independent predictors of AIS (Supplementary Table S2). Therefore, s-AP3D1-Ab marker may discriminate a certain type, if not all, of atherosclerosis caused by HT or DM, leading to the development of AIS and CVD.

AP3D1 is a subunit of the AP3 adaptor-like complex^35^, which is expressed in the ubiquitous AP3 complex and also in the neuronal form^36^. AP3 is a heterotetrameric adaptor protein involved in the biogenesis of lysosome-related organelles, such as platelet-dense bodies. Mouse mutants of the null AP3D1 allele were reported to show abnormal bleeding due to the absence of a storage pool of dense platelet particles, raising the possibility that AP3D1 functions as a thrombogenic regulator through platelet function^37^. Platelets have been identified to play a significant role in hemostatic and thrombotic processes, where abnormal platelet adhesion/activation can lead to the formation of clots (thrombosis)^38^. Thrombosis is known to be closely associated with atherosclerosis^39^. Hirokawa et al.^40^ reported that AP3D1-DOT1L-SF3A2 was identified as a new susceptibility locus for myocardial infarction (MI) by European genome-wide association studies (GWAS), which is consistent with our observation that s-AP3D1-Abs levels were significantly higher in patients with CVD including MI (Fig. 3a, Table 3). On the other hand, Xiao et al. reported that angiotensin II facilitated the binding of AP3D1 with beta-arrestin, which resulted in the activation of AP3D1 as a scaffold protein^41^. Angiotensin II plays a key role in the pathogenesis of HT^42–45^, leading to endothelial dysfunction and atherosclerosis^46,47^. AP3D1 can possibly mediate angiotensin II-induced HT and atherosclerosis. This was compatible with the results that the s-AP3D1-Ab levels were significantly associated with HT (*P* < 0.0001) (Table 6) and BP (*P* < 0.001) (Table 7).

HT is also known as a risk factor for various types of cancers^48–51^. Previous reports have proved that angiotensin II is associated with cancer development^52,53^. Angiotensin II is a major upstream regulator of cancer cachexia^54^ and can further stimulate angiogenesis and tumor growth of breast cancer^55,56^. Thus, AP3D1 could also mediate angiotensin II-induced carcinogenesis. Based on the results, s-AP3D1-Abs levels were elevated in cancer patients (Fig. 5).

The progression of atherosclerosis and cancer often takes several years or more and, in the early stages, is sometimes accompanied by low levels of tissue destruction, which can lead to leakage of proteins from the cells. During this repeated leakage of proteins, even low levels of antigens can induce amplified expression of the antibodies^57^. Thus, antibody markers are deemed more sensitive than antigen markers and may be useful for the early diagnosis of solid cancers including ESCC and CRC. s-AP3D1-Ab marker was closely associated with max IMT, an index of atherosclerosis, which then leads to the onset of AIS and AMI. Thus, predicting these onsets using s-AP3D1-Ab marker is possible. In fact, the results of JPHC cohort analysis indicated that the antibody marker against AP3D1 is useful in predicting the onset of AIS (Table 8). Atherosclerotic AIS and AMI and cancer have been identified as the leading causes of death; thus, the s-AP3D1-Ab marker would be highly useful to reduce its mortality.

As antihypertensive agents, statins, and antiplatelet agents are generally known to prevent the pathogenesis of atherosclerosis^58–60^, the potential modulatory effects of these drugs on s-AP3D1-Ab levels must be considered. Second, as the controls were healthy volunteer donors, potential confounding factors between patients with atherosclerosis, cancers, and controls (age, BMI, HT, DM, and hyperlipidemia) were not adjusted in the analysis of this study. Lastly, physiological testing, such as baPWV, or coronary artery calcification, was not performed to evaluate atherosclerosis in subjects subjected to the s-AP3D1-Ab analysis. Nevertheless, these tests might be expected to confirm the results of this study. The study population included only Japanese patients; thus, further studies are required in patients who are not taking drugs that can affect atherosclerosis and in other ethnic groups. Development of more biomarkers for the early diagnosis of atherosclerotic diseases and early detection of the development of tumors may improve the quality of life.

## Supplementary Information

Supplementary Information 1.

Supplementary Information 2.

## Data Availability

The datasets used and/or analyzed during the current study are available from the corresponding author on reasonable request.
