# Supplementary material for: Serum anti-AP3D1 antibodies are risk factors for acute ischemic stroke related with atherosclerosis
Source: Sci Rep. 2021 Jun 29;11:13450. doi: 10.1038/s41598-021-92786-9 (PMC8242008; doi:10.1038/s41598-021-92786-9)
Supplement: Supplementary file 1 — Supplementary Information 1. [file 41598_2021_92786_MOESM1_ESM.pptx]

## Slide 1
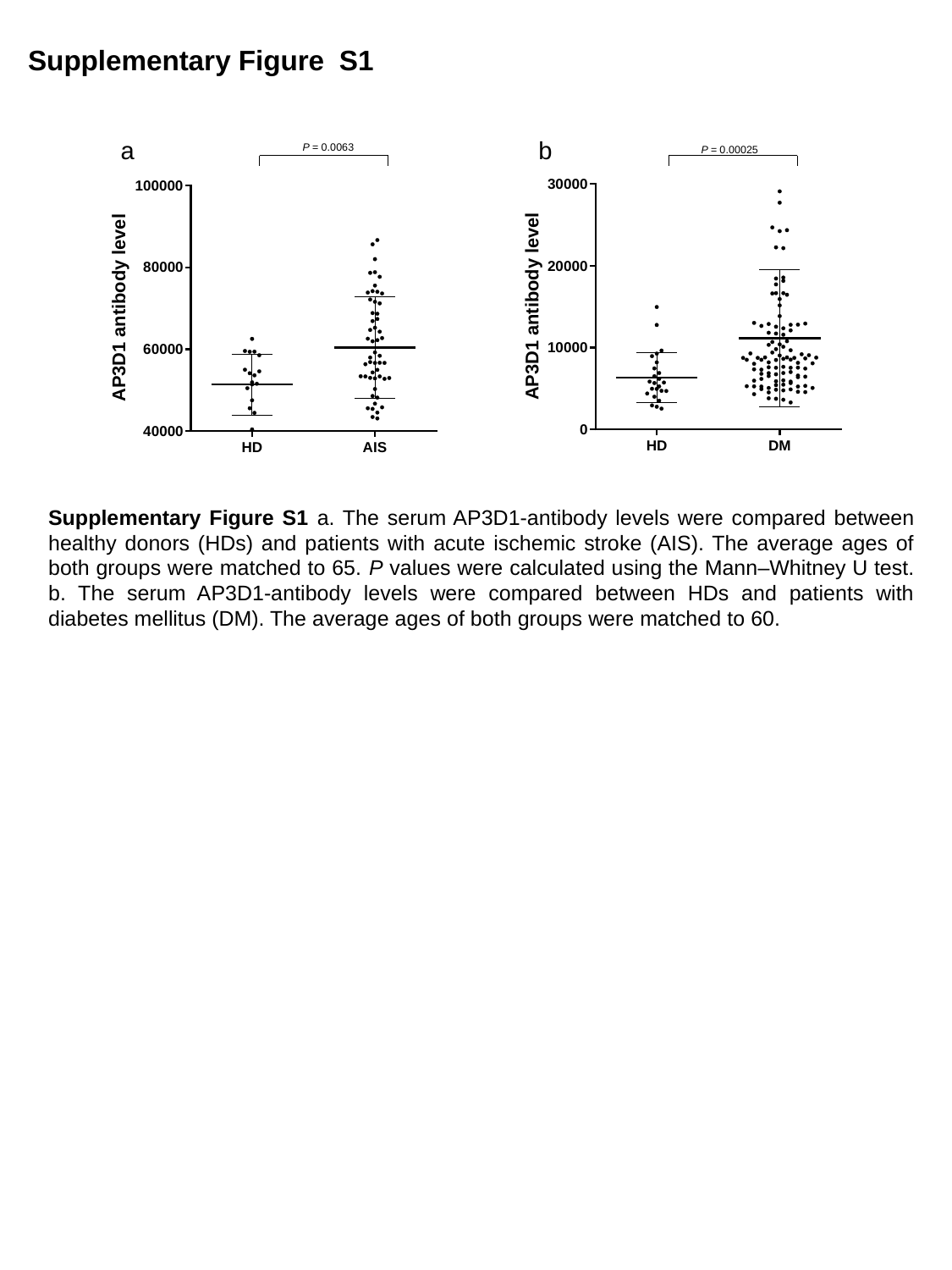

Supplementary Figure S1
a
P = 0.0063
b
P = 0.00025
Supplementary Figure S1 a. The serum AP3D1-antibody levels were compared between healthy donors (HDs) and patients with acute ischemic stroke (AIS). The average ages of both groups were matched to 65. P values were calculated using the Mann–Whitney U test. b. The serum AP3D1-antibody levels were compared between HDs and patients with diabetes mellitus (DM). The average ages of both groups were matched to 60.
