# Supplementary material for: Serum anti-AP3D1 antibodies are risk factors for acute ischemic stroke related with atherosclerosis
Source: Sci Rep. 2021 Jun 29;11:13450. doi: 10.1038/s41598-021-92786-9 (PMC8242008; doi:10.1038/s41598-021-92786-9)
Supplement: Supplementary file 2 — Supplementary Information 2. [file 41598_2021_92786_MOESM2_ESM.docx]

**Serum anti-****AP3D1 antibodies are risk factors for acute ischemic stroke related with atherosclerosis**

Shu-Yang Li^1^, Yoichi Yoshida^1,2^, Eiichi Kobayashi^1,2^, Masaaki Kubota^1^, Tomoo Matsutani^1^, Seiichiro Mine^1,3,4^, Toshio Machida^1,4,5^, Yoshiro Maezawa^6^, Minoru Takemoto^6,7^, Koutaro Yokote^6^, Yoshio Kobayashi^8^, Hirotaka Takizawa^9^, Mizuki Sata^10,11^, Kazumasa Yamagishi^10^, Hiroyasu Iso^12^, Norie Sawada^13^, Shoichiro Tsugane^13^, Sohei Kobayashi^14,15^, Kazuyuki Matsushita^14^, Fumio Nomura^16^, Hisahiro Matsubara^17^, Makoto Sumazaki^18^, Masaaki Ito^18^, Satoshi Yajima^18^, Hideaki Shimada^18^, Katsuro Iwase^19,^ Hiromi Ashino^19^, Hao Wang^19,20^, Kenichiro Goto^19^, Go Tomiyoshi^19,21^, Natsuko Shinmen^19,21^, Rika Nakamura^19,21^, Hideyuki Kuroda^21^, Yasuo Iwadate^1,2^ & Takaki Hiwasa^1,2,18,19,20^*

^1^Department of Neurological Surgery, Graduate School of Medicine, Chiba University, Chiba 260-8670; ^2^Comprehensive Stroke Center, Chiba University Hospital, Chiba 260-8677; ^3^Department of Neurological Surgery, Chiba Prefectural Sawara Hospital, Chiba 287-0003; ^4^Department of Neurological Surgery, Chiba Cerebral and Cardiovascular Center, Chiba 290-0512; ^5^Department of Neurosurgery, Eastern Chiba Medical Center, Chiba 283-8686; ^6^Department of Endocrinology, Hematology and Gerontology, Graduate School of Medicine, Chiba University, Chiba 260-8670; ^7^Department of Diabetes, Metabolism and Endocrinology, School of Medicine, International University of Health and Welfare, Chiba 286-8686; ^8^Department of Cardiovascular Medicine, Graduate School of Medicine, Chiba University, Chiba 260-8670, Japan; ^9^Port Square Kashiwado Clinic, Kashiwado Memorial Foundation, Chiba 260-0025, Japan; ^10^Department of Public Health Medicine, Faculty of Medicine, University of Tsukuba, Tsukuba 305-8575, Japan; ^11^Department of Preventive Medicine and Public Health, Keio University School of Medicine, Tokyo 160-8582, Japan; ^12^Department of Public Health, Social Department of Social and Environmental Medicine, Graduate School of Medicine, Osaka University, Osaka 565-0871, Japan; ^13^Epidemiology and Prevention Group, Center for Public Health Sciences, National Cancer Center, Tokyo 104-0045, Japan; ^14^Department of Laboratory Medicine & Division of Clinical Genetics, Chiba University Hospital, Chiba 260-8677, Japan; ^15^Department of Medical Technology & Sciences, School of Health Sciences at Narita, International University of Health and Welfare, Chiba 286-8686, Japan; ^16^Division of Clinical Genetics, Chiba Foundation for Health Promotion & Disease Prevention, Chiba 261-0002, Japan; ^17^Department of Frontier Surgery, Graduate School of Medicine, Chiba University, Chiba 260-8670, Japan; ^18^Department of Gastroenterological Surgery and Clinical Oncology, Toho University Graduate School of Medicine, Tokyo 143-8541, Japan; ^19^Department of Biochemistry and Genetics, Graduate School of Medicine, Chiba University, Chiba 260-8670, Japan; ^20^Department of Anesthesia, The First Affiliated Hospital, Jinan University, Guangzhou, Guangdong 510632, P.R. China; ^21^Medical Project Division, Research Development Center, Fujikura Kasei Co., Saitama 340-0203, Japan

*Correspondence: Takaki Hiwasa, Department of Neurological Surgery, Graduate School of Medicine, Chiba University, Inohana 1-8-1, Chuo-ku, Chiba 260-8670, Japan

E‑mail: [hiwasa_takaki@faculty.chiba-u.jp](mailto:hiwasa_takaki@faculty.chiba-u.jp)

**Supplementary Table S1.** Baseline characteristics of the study subjects

| Parameter | HD | AIS | cCI | TIA | asymptCI | DSWMH | Other disease |
| --- | --- | --- | --- | --- | --- | --- | --- |
| Total number | 139 | 226 | 57 | 43 | 17 | 121 | 30 |
| Age (years) | 51.77± 12.72 | 77.08± 11.15 | 72.42±8.89 | 68.56± 12.27 | 66.29± 11.90 | 66.29± 10.30 | 65.63± 18.20 |
| Male sex | 87 (62.6%) | 129 (57.1%) | 18 (68.4%) | 23 (53.5%) | 12 (70.6%) | 71 (58.7%) | 18 (60.0%) |
| Hypertension | 41 (29.5%) | 182 (80.5%) | 48 (84.2%) | 34 (79.1%) | 12 (70.6%) | 63 (52.1%) | 17 (56.7%) |
| Diabetes | 8 (5.8%) | 59 (26.1%) | 20 (35.1%) | 12 (27.9%) | 5 (29.4%) | 17 (14.0%) | 2 (6.7%) |
| Hyperlipidemia | 28 (20.1%) | 54 (23.9%) | 24 (42.1%) | 15 (34.9%) | 4 (23.5%) | 42 (34.7%) | 9 (30.0%) |
| CVD | 1 (0.7%) | 22 (9.7%) | 2 (3.5%) | 3 (7.0%) | 0 (0%) | 7 (5.8%) | 1 (3.3%) |
| Obesity (BMI ≥25) | 43 (30.9%) | 56 (24.8%) | 5 (8.8%) | 12 (27.9%) | 2 (11.8%) | 23 (19.0%) | 9 (30.0%) |
| Smoking | 58 (41.7%) | 113 (50%) | 28 (49.1%) | 24 (55.8%) | 10 (58.8%) | 54 (44.6%) | 15 (50.0%) |

AIS, AIS, acute ischemic stroke; TIA, transient ischemic attack; HD, healthy donor; cCI, chronic cerebral infarction; asymptCI, asymptomatic cerebral infarction; DSWMH, deep and subcortical white matter hyperintensity; CVD, cardiovascular disease.

**Supplementary Table S2.** Logistic regression analysis of predictive factors for AIS (total no., 365; no. of events, 226)

|  | Univariate analysis | Multivariate analysis | | | |
| --- | --- | --- | --- | --- | --- |
|  | *P* value |  | 95% CI |  | *P* value |
| Age, years (≥60) | **<0.0001** |  | 7.85–32.40 |  | **<0.0001** |
| Male | 0.324 |  |  |  |  |
| HT | **<0.0001** |  | 2.36–8.19 |  | **<0.0001** |
| DM | **<0.0001** |  | 2.39–20.40 |  | **<0.001** |
| Lipidemia | 0.442 |  |  |  |  |
| CVD | **<0.001** |  | 0.48–30.30 |  | 0.204 |
| Obesity (BMI ≥25) | 0.176 |  |  |  |  |
| Smoking | 0.13 |  |  |  |  |
| AP3D1-Ab (>7082) | **<0.0001** |  | 0.66–2.56 |  | 0.445 |

Significant associations (*P* <0.05) are marked in bold font. AP3D1-Ab cutoff value was 7082 based on ROC curve analysis. CI, confidence interval. Significant associations (*P* <0.05) are marked in bold font.

**Supplementary Table S3.** Power calculation of the subjects

|  | AIS | TIA | DM | CVD | CKD type 1 |
| --- | --- | --- | --- | --- | --- |
| Tail (s) | Two | Two | Two | Two | Two |
| Effect size d | 0.4756515 | 0.6659121 | 0.920358 | 0.9701425 | 1.102426 |
| α err prob | 0.05 | 0.05 | 0.05 | 0.05 | 0.05 |
| Sample size of HD | 123 | 77 | 81 | 78 | 82 |
| Sample size group 2 | 158 | 123 | 275 | 100 | 145 |
| **Power calculation (1-β err prob)** | **0.9762618** | **0.9953412** | **0.9999999** | **0.9999952** | **1** |
|  |  |  |  |  |  |
|  | CKD type 2 | CKD type 3 | ESCC | CRC |  |
| Tail (s) | Two | Two | Two | Two |  |
| Effect size d | 1.4980538 | 0.8688712 | 1.3409568 | 0.6651901 |  |
| α err prob | 0.05 | 0.05 | 0.05 | 0.05 |  |
| Sample size of HD | 82 | 82 | 64 | 64 |  |
| Sample size of patients | 32 | 123 | 64 | 64 |  |
| **Power calculation (1-β err prob)** | **0.9999999** | **0.9999798** | **1** | **0.9619756** |  |

Using the Software: Gpower 3.1 to analysis post hoc: Compute achieved power with the t tests - Means: Difference between two independent means (two groups).
